# Association of state postpartum depression legislation and maternal mortality in the United States

**DOI:** 10.3389/fpubh.2026.1625860

**Published:** 2026-03-06

**Authors:** Jonné McCoy-White, JohnBosco Chika Chukwuorji, Sania Farooq, Alanna Foulon, Quincy Frisbey, Haneen Hammad, Takeya Harris, Amy M. Loree, Caron Zlotnick, Jennifer E. Johnson

**Affiliations:** 1 Charles Stewart Mott Department of Public Health, College of Human Medicine, Michigan State University, Flint, MI, United States; 2 Center for Health Policy and Health Services Research, Henry Ford Health, Detroit, MI, United States; 3 Women and Infants Hospital and Department of Psychiatry and Human Behavior, Brown University, Providence, RI, United States; 4 Department of Psychiatry and Mental Health, University of Cape Town, Cape Town, South Africa

**Keywords:** maternal health, maternal health policy, maternal mental health, maternal mental health policy, postpartum depression

## Abstract

**Objective:**

State legislation addressing postpartum depression varies widely across the U.S. and may play a critical role in shaping access to mental health care and supports that influence state level maternal mortality outcomes. This study examined the relationship between U.S. state legislation on postpartum depression (PPD) and state-level maternal mortality rates.

**Methods:**

Data from PPD-related legislation in 32 states were reviewed and were categorized based on their scope and funding. State-level maternal mortality data was obtained from the CDC, and uninsured rates from the U.S. Census Bureau's American Community Survey.

**Results:**

States with highest policy quality PPD legislation, especially those with funding, and with more PPD-related legislation had lower maternal mortality rates, even after adjusting for uninsured rates. In regression analyses, uninsured rate explained 23% of the variance in maternal mortality; number of PPD policies explained 13%, and PPD policy quality explained 12%.

**Conclusions:**

Higher quantity and highest quality of PPD-related state legislation are associated with lower state-level maternal mortality.

## Background

This paper assesses the relationship between the presence and quality of U.S. state legislation related to postpartum depression (PPD) and state-level maternal mortality. PPD is a significant public health issue, particularly among low-income women (1, 2). Approximately one in seven women experience PPD (3). Untreated PPD can have severe and lasting consequences for mother and infant, including maternal increased risk of suicide (4), compromised functional status (5–7) and social functioning (4, 8–15), less maternal time in positive activities with infant (14), and less maternal infant sensitivity (16). For infants, the effect of untreated PPD are also severe and lasting, with poorer cognitive and language development from infancy to late childhood (17–20). Furthermore, the cost of health and social care is much higher in women with PPD compared to those without PPD (21). Although not counted in maternal mortality rates (22, 23), suicide and intimate partner violence are among leading causes of death during the perinatal period (24, 25). Both are associated with PPD.

PPD occurrence in low-income mothers is almost double in comparison to their higher income counterparts (22). In addition, the consequences of PPD can be more severe for low-income women. The negative effects of untreated PPD on mother-infant interactions, maternal caregiving, infant development, and child language development appear to be intensified by socioeconomic adversity (16, 17, 26). Among low-income women, the higher prevalence of PPD, the reduced likelihood of treatment, and the impact of untreated PPD on impaired development in children (27, 28) leads to health disparities in children of these affected mothers (29). The robust evidence linking postpartum depression to adverse maternal and child outcomes highlights PPD not only as a clinical concern but as a policy-relevant determinant of intergenerational health.

There are effective approaches to prevent, screen, and treat PPD. In 2019, the US Preventive Services Task Force (USPSTF) issued a recommendation indicating that it is possible to prevent PPD through counseling interventions (30–32). For example, the Reach Out, Stand Strong, Essentials for New Mothers (ROSE) program consists of four classes during pregnancy and a postpartum check-in. ROSE has been tested in four randomized controlled trials among low-income women in community prenatal settings and found to prevent half of PPD cases (33–36). There are also well-established guidelines for screening for PPD (37). These include screening for depression at least twice during pregnancy, at postpartum visits, and at pediatric well-infant/child visits and well-woman visits, using validated screening tools (38). If PPD is identified, effective psychosocial (such as interpersonal psychotherapy or intensive postpartum home visits) (39) and pharmacological (38) treatments have been identified.

In the past 20 years, many states have started passing legislation to help address PPD. New Jersey was the first state to enact a state law mandating PPD screening and education for mothers and new parents in 2006. Other states have mandated education and awareness or issued gubernatorial proclamations about the importance of PPD (1). State legislation addressing perinatal mental health has ranged from patient education requirements, depression screening requirements, task force requirements, and/or public awareness campaigns. Some of these efforts have included budgetary or fiscal appropriations while others have not (40). The relationship between such legislation and maternal mortality has not yet been examined.

The most recent data from the U.S. Centers for Disease Control and Prevention revealed that in 2023, 669 women died of pregnancy-related causes (during pregnancy or within 42 days of the end of pregnancy/childbirth), a decrease from 817 in 2022 (41). The CDC counts include only pregnancy-related deaths. Pregnancy-associated deaths (which are defined more broadly, including suicide, overdose, and intimate partner violence up to 1 year post-partum) are excluded from the CDC's count (41).

## Methods

### Procedures

This brief analyzes enacted legislation and resolutions related to PPD in 32 U.S. states. The legislative review was conducted as part of a national implementation trial (42) of the evidence-based ROSE PPD prevention program, which worked with agencies in the 32 states. The current analysis is a secondary analysis of the trial's legislative review data. In predicting state-level maternal mortality, our analyses also control for state level uninsured rates, a well-known predictor of maternal mortality (39). Mean maternal mortality rates in the 32 included states (23.3 per 100,000 live births) was similar to those in the other 18 states plus the District of Columbia (23.9 per 100,000 live births). The 32 states included had more births per state on average (*n* = 495,135 births from 2018–2022) than did those that were not included (*n* = 138,980 from 2018–2022). The included states contained 87% of births in the U.S. from 2018–2022.

## Measures

### PPD legislation

Our legal and policy review included laws, resolutions, and codes pertaining to PPD or maternal mental health enacted through 2018. We gathered state policy data through four methods: Lexis Nexis, Open States, state legislature websites, and a general Google search. Lexis Nexis, a legal database, encompasses records from both U.S. and international jurisdictions. Open States, a bill-tracking website, monitors legislative activities across all 50 states. Using the generic search bar at the top of the legislative website or the search bars specific to bills, laws, or codes on the legislative website, the search terms “postpartum depression,” “perinatal mental health,” and “maternal mental health” were utilized to populate applicable bills, codes, resolutions, and laws. Each individual state legislative website varied in their design and usability relative to searchability. Dependent upon each state legislature's filtering mechanisms, we replicated these search terms for all available session years up until the end of 2018. From the populated search results, each applicable bill, law, code, and resolution was individually reviewed and coded. Relevant bills that populated from these searches were reviewed to ensure that they resulted in enacted legislation to be eligible for inclusion. The search keywords included: “postpartum depression,” “perinatal mental health,” “maternal mental health,” and for the Google search, “(specific state) postpartum depression legislation.” For e.g., “Michigan postpartum depression legislation.” Throughout our coding process, only enacted state legislation was considered. We counted the total number of state laws related to PPD. In addition, states were categorized by highest policy quality as described below. Similar indices have been used in other policy analyses (43).

#### 0: No enacted state legislation regarding postpartum depression

States that were coded “0” may have had preliminary bills related to PPD, but these bills did not result in any codified law or resolution.

#### 1: Awareness-related legislation

Score of one refers to awareness-related legislation, defined as any enacted legislation that brings awareness to PPD or perinatal/maternal mental health. Examples of this type of legislation include mandating a “maternal mental health awareness month” or establishing a task force to investigate PPD without any services to support or educate about the condition.

#### 2: Legislation mandating postpartum depression education or services but without funding attached

Score of two refers to legislation mandating PPD education and services commonly seen as mandated screening, referral, treatment, and/or provider training, but without any budgetary and fiscal appropriations for the services through Medicaid or other sources.

#### 3: Legislation mandating postpartum depression education or services that also provided funding for these activities

Score of three refers to legislation with a budget or monetary fiscal appropriation attached to the PPD education or services.

**State-level uninsured rate** was taken by overall percent uninsured by state was collected from the Census Bureau's American Community Survey and analyzed by KFF (44).

**State-level maternal mortality rate** over 2018–2022 used Centers for Disease Control and Prevention (CDC) data (45) for pregnancy-related maternal mortality (46). Because maternal deaths are rare events, annual state-level data can show large year-to-year fluctuations that do not reflect true trends. Therefore, we used the CDC's 5-year average to create more stable and reliable rates.

### Statistical analyses

We conducted multiple linear regression analyses to predict pregnancy-related maternal mortality rates (averaged over 2018–2022) based on the overall percent of uninsured individuals in the state (2018–2022 Uninsured %), number of policies enacted through 2018, and the highest policy score assigned to legislation in the state through 2018 (highest policy quality). We had planned to then enter all predictors simultaneously, but number of policies and policy quality were highly correlated (*r* = 0.72, *p* < 0.001), each explaining 52% of the variance in the other. Therefore, we analyzed effects of number of policies and highest policy quality separately, controlling for uninsured rates.

## Results

Table 1 describes uninsured rate 2018–2022, total number of PPD-related policies through 2018, highest policy quality through 2018, and pregnancy-related maternal mortality rate (2018–2022) by state. Texas had the highest uninsured rate (17.7%) but also the highest number of PPD policies (*n* = 13). Mississippi had the highest 5-year maternal mortality rate.

**Table 1:** Uninsured rates, highest policy quality, number of policies, and maternal mortality rate by state.

**Clinic**	**Uninsured rate % 2018–2022**	**Highest policy quality**	**Number of PPD policies enacted through 2018**	**Maternal mortality rate (per 100,000 live births) 2018–2022**
Arizona	0.11	0	0	38.6
California	0.08	2	7	10.5
Colorado	0.08	1	1	16.0
Florida	0.12	3	2	24.1
Georgia	0.13	0	0	32.1
Hawai	0.04	0	0	16.1
Illinois	0.07	2	5	18.1
Indiana	0.08	1	1	30.9
Iowa	0.05	0	0	19.5
Kansas	0.09	0	0	22.8
Kentucky	0.06	0	0	34.6
Louisiana	0.08	0	0	37.3
Maryland	0.06	3	4	21.3
Massachusetts	0.03	3	9	16.4
Michigan	0.05	1	2	19.1
Minnesota	0.04	3	5	12.3
Mississippi	0.12	0	0	39.1
Missouri	0.09	0	0	23.8
New Jersey	0.07	3	1	26.0
New Mexico	0.09	3	4	28.0
New York	0.05	2	1	22.4
North Carolina	0.10	0	0	26.7
North Dakota	0.07	3	5	21.6
Ohio	0.06	0	0	24.5
Oregon	0.07	2	4	16.6
Pennsylvania	0.05	1	2	17.5
Rhode Island	0.05	2	3	17.5
South Carolina	0.10	0	0	32.3
Texas	0.18	3	13	28.2
Utah	0.09	3	2	15.5
Virginia	0.08	1	1	32.7
Wisconsin	0.05	0	0	13.2

Highest policy quality was coded as follows: 3 (PPD policy with $ and services); 2 PPD policy with services but no $); 1 (an awareness related PPD policy); 0 (no PPD-related policy).

Table 2 presents results including number of policies and highest policy quality in separate linear regression analyses, controlling for uninsured rate. In the models, higher uninsured rates were associated with higher maternal mortality rate (*p* < 0.001), accounting for 23% of the variance in maternal mortality rate. In the first model, higher number of policies was associated with lower maternal mortality rate, accounting for 13% of the variance in maternal mortality rate (changes in *R*^2^ in the regression model). In the second model, highest PPD policy quality within each state was associated with lower maternal mortality rate, also accounting for 12% of the variance in maternal mortality rate. On average, states' maternal mortality rates lowered by one in 100,000 births for each additional PPD-related policy that was enacted (see *B* in Table 2). On average, states with legislation mandating PPD services and providing funding for them had maternal mortality rates that were seven in 100,000 lower than those without PPD-related legislation.

**Table 2:** Separate regressions predicting state-level pregnancy-related maternal mortality rates by PPD-related policy quality and number of policies, controlling for percent uninsured, in 32 states.

**Predictor**	***B***	**β**	**95% CI (*B*)**	**Predictor**	***B***	**β**	**95% CI (*B*)**
% Uninsured	127.59	0.51^**^	51.42, 203.73	% Uninsured	119.51	0.48^**^	43.36, 195.66
Number of policies	−0.89	−0.36^*^	−1.66, −0.13	Highest policy quality	−2.06	−0.35^*^	−3.89, −0.23

^*^p < 0.05; ^**^p < 0.01.

Highest policy quality was coded as follows: 3 (PPD policy with $ and services; 2 PPD policy with services but no $); 1 (an awareness related PPD policy); 0 (no PPD-related policy).

R2 = 0.23 (% Uninsured), 0.13 (Number of policies) and.12 (PPD Policy quality).

### Policy implications

Our findings indicate that U.S. states with higher quantity and quality of PPD policies had lower pregnancy-related maternal mortality rates. These relationships held even after adjusting for uninsured rate, suggesting that dedicated PPD legislation may contribute to or reflect effective maternal health strategies. Uninsured rates accounted for 23% of the variation in maternal mortality in each model, with number of PPD policies and PPD policy quality accounting for another 13% in the models. Our finding that uninsured rates accounted for a large minority of the variation in state-level maternal mortality is consistent with other work showing the importance of insurance coverage for maternal health. For example, Medicaid expansion under the Affordable Care Act was linked to seven fewer maternal deaths per 100,000 live births overall, and 16 fewer deaths per 100,000 among Black women (47). We found that having policies mandating PPD services and funding (vs. no PPD policies) was associated with seven fewer maternal deaths per 100,000 live births on average, an effect nearly as large as that of Medicaid expansion.

There are several reasons PPD policies and pregnancy-related maternal mortality (which does not include suicide, partner violence, or overdose) may be associated with each other. Because the study is correlational and not causal, PPD policies may influence maternal mortality, maternal mortality may influence PPD policies, or unrelated factors could be contributing to both. For example, some legislated resources address both PPD and maternal health, such as community health workers, doulas, home visiting, and other supportive services, which have been found to improve maternal health outcomes and save lives (48). It is also possible that states with a stronger and more organized maternal health workforce are more able to advocate for PPD and other maternal health legislation. In other words, states that prioritize maternal health may be enacting more PPD legislation. Finally, some states (or large health systems within the states) have made maternal health a priority, resulting in both more and better PPD legislation and lower maternal mortality.

In this sample, number of policies and highest policy quality were highly correlated (*r* = 0.72), each explaining more than half the variance in the other. In other words, states with more policies were more likely to have at least one high-quality policy than states with fewer policies.

Although not directly examined in this paper, some have proposed (49) that policies mandating and providing funding for maternal health services may help reduce health disparities by improving access to screening and treatment for women who are historically underserved, including racial and ethnic minority women, low-income women, and those living in rural areas. Due to the intersection of these social and structural disadvantages, experts have argued that comprehensive and funded legislation could promote more equitable maternal health outcomes across societal, historical, and institutional processes that perpetuate inequities (49).

Strengths of this research include a careful legislative review and a novel question. Limitations include: (1) use of only 32 states (containing 87% of U.S. births) in the analysis; (2) a cross-sectional design which limits causal conclusions about relationships between PPD policy mechanisms and maternal mortality outcomes; (3) use of pregnancy-related mortality data, which excludes deaths from homicide, overdose, and suicide, which are mostly closely associated with untreated PPD. In addition, other variables that were not included, such as states' investment in maternal health, racial disparities in access to care, and political climate, may influence adoption of PPD legislation and maternal mortality rates. Because this is an observational, cross-sectional study, our findings reflect associations and cannot be interpreted as causal effects. Future analyses of longitudinal patterns (e.g., by using two-way fixed effects to examine whether maternal mortality falls after PPD-related legislation is enacted) could be informative. In addition, analyses that include pregnancy-associated deaths (e.g., suicide, overdose, and homicide) rather than pregnancy-related deaths only (as defined by the CDC), that examine reach and implementation of legislated actions, that examine socioeconomic and social factors, and/or that incorporate qualitative analyses may provide a deeper understanding of the effects of state PPD policy on maternal health outcomes.

### Actionable recommendations

Medicaid expansion coverage to help reduce the uninsured rate is critical, as uninsured people account for 23% of the variation in maternal mortality in this study. Furthermore, support for more and higher quality state PPD policies (e.g., with mandates, funding).

## Conclusion

States with strong, well-funded postpartum depression (PPD) policies and lower uninsured rates tend to have lower maternal mortality rates. The findings in this policy brief can be of use to policymakers, practitioners, and public health professionals in advocating for and implementing comprehensive maternal health policies that include dedicated funding to ensure effective implementation.
